# Leveraging knowledge graphs and large language models for integrating molecular variants and clinical insights in COVID-19 research

**DOI:** 10.1016/j.bsheal.2025.12.003

**Published:** 2025-12-20

**Authors:** Jiaxin Yang, Fushuai Zhang, Ruifang Cao, Yingying Chen, Yiping Chen, Yuxin Chen, Yixue Li, Guoping Zhao, Ying Wang, Yunchao Ling, Guoqing Zhang

**Affiliations:** a School of Life Science, Hangzhou Institute for Advanced Study, University of Chinese Academy of Sciences, Hangzhou 310024, China; b Bio-Med Big Data Center, Shanghai Institute of Nutrition and Health, Chinese Academy of Sciences, Shanghai 200031, China; c State Key Laboratory of Microbial Technology, Institute of Microbial Technology, Shandong University, Qingdao 266237, China; d Shanghai Institute of Immunology, Department of Microbiology and Immunology, Shanghai Jiao Tong University School of Medicine, Shanghai 200025, China; e Guangzhou National Laboratory, Guangzhou 510005, China; f Department of Oto-Rhino-Laryngology, Institute of Rare Diseases, Frontiers Science Center for Disease-related Molecular Networks, West China Hospital, Chengdu 610213, China; g Shanghai Institute of Virology, Shanghai Jiao Tong University School of Medicine, Shanghai 201318, China

**Keywords:** Coronavirus disease 2019 (COVID-19), Severe acute respiratory syndrome coronavirus 2 (SARS-CoV-2), Knowledge graph, Large language model, Variant risk assessment, Mutation function mapping, Early warning system, Vaccine design

## Abstract

The relentless emergence of severe acute respiratory syndrome coronavirus 2 (SARS-CoV-2) variants continues to challenge global health, as high mutation rates and complex pathogenicity obscure molecular mechanisms and impede clinical progress. Despite extensive research across viral evolution, structural biology, immunology, diagnostics, and therapeutics, the resulting vast and rapidly outdated literature has widened the gap between fundamental discovery and medical application. Here, we systematically mined 439,724 coronavirus disease 2019 (COVID-19) publications using fine-tuned large language models to extract and distill knowledge across nine domains: antibodies, vaccines, serology, biochemistry, therapeutics, clinical presentation, risk factors, biomarkers, and diagnostics. These insights were integrated into a unified graph of 1,427,596 triples (CoVAR-KG). Covering 90 % of known spike-protein variant sites, our knowledge graph forges molecular-to-clinical links that reveal how specific mutations influence antigenicity, transmissibility, and treatment response. By resolving data fragmentation, this resource accelerates target identification and streamlines hypothesis generation. Building on CoVAR-KG, we developed COVID-19 variant risk watcher (CVRW), an early-warning framework that quantifies the threat of emerging variants for real-time surveillance. Coupling the graph with retrieval-augmented GPT-4o enables rapid and in-depth comparisons of variant functionality and immune escape potential. These integrative tools furnish timely insights for vaccine design, therapeutic optimization, and pandemic preparedness, establishing a versatile platform for combating current and future viral threats.

## Highlights

• **Scientific question** This study addresses the challenge of systematically linking severe acute respiratory syndrome coronavirus 2 (SARS-CoV-2) spike-protein substitutions with clinical, immunological, and therapeutic outcomes for functional risk assessment of emerging variants.
• **Evidence before this study** Existing coronavirus disease 2019 (COVID-19) databases and analytical frameworks are fragmented across molecular, immunological, and clinical domains, limiting integrative interpretation of mutation-driven functional effects.
• **New findings** We curated and mined 439,724 COVID-19 studies to construct a 1-million-node knowledge graph (CoVAR-KG) spanning nine biomedical domains. Building on this resource, we developed the COVID-19 Variant Risk Watcher (CVRW), which combines graph-based retrieval with GPT-4o to forecast the World Health Organization (WHO) variant classifications in near real time.
• **Significance of the study** This integrative framework enables interpretable, literature-grounded evaluation of mutation-induced changes in antigenicity, transmissibility, and immune escape, providing a scalable foundation for genomic surveillance and public health decision-making.

## 1 Introduction

Since its emergence in late 2019, coronavirus disease 2019 (COVID-19) has posed an unprecedented global threat to public health and biomedical research [1], [2]. Severe acute respiratory syndrome coronavirus 2 (SARS-CoV-2) combines a high mutation rate with potent pathogenicity and rapid transmission, continually undermining epidemic control, vaccine efficacy, and therapeutic strategies [3], [4], [5]. The relentless rise of novel variants accelerates immune escape and diminishes the protective value of existing vaccines and monoclonal antibodies [3], [5], while the rapid pace of viral evolution renders critical insights obsolete before they can inform clinical practice or policy [5]. Accurately predicting the functional consequences of each emerging strain is therefore essential for guiding timely interventions. Early machine learning (ML) and deep learning (DL) studies demonstrated this potential: spike mutations such as L452 and N501 were computationally predicted in mid-2020 [6], with subsequent analyses revealing mechanisms of enhanced infectivity [6], antibody escape [7], and even forecasted the dominance of Omicron BA.2, BA.4, and BA.5 lineages ahead of World Health Organization (WHO)‌ confirmation [8], [9].

The scientific response to COVID-19 spans epidemiology [10], [11], [12], [13], clinical trials [14], [15], molecular pathogenesis [16], [17], [18], therapeutics [19], [20], and vaccine design [21], [22]. Yet deep disciplinary divides persist: virologists elucidate viral biology, while clinicians focus on patient outcomes, diagnostics, and treatment efficacy, and few platforms bridge these realms effectively [22], [23]. Although knowledge graphs such as KG-COVID-19 [24], the COVID-19 pathophysiology graph [25], and the COVID-19 vaccine graph [26] integrate domain-specific data, they often overlook the full spectrum of mutation-to-clinical-outcome relationships. To overcome these gaps, we have built a comprehensive knowledge graph that unites molecular mechanisms, clinical findings, and therapeutic insights into a single, navigable resource.

To address these challenges, we built a comprehensive, SARS-CoV-2–focused knowledge graph (CoVAR-KG, available at https://www.biosino.org/CoVAR-KG), that unites molecular mechanisms with clinical outcomes into a single, navigable framework. By mining 439,724 COVID-19 publications, we extracted and harmonized over 1 million knowledge triples covering monoclonal antibodies, vaccines, therapeutics, and the functional impacts of spike-protein substitutions on pathogenicity, transmissibility, and immunogenicity. We complemented this graph with a curated COVID-19 annotation dataset—capturing mutation features, immune responses, therapeutic targets, and vaccine candidates—and developed a fine-tuned, domain-specific large language model (LLM) using model distillation, chain-of-thought reasoning, and few-shot learning to streamline large-scale graph construction. Covering 90 % of known spike-protein variant sites, the graph successfully predicted the increased infectivity and immune evasion of the KP.2 subvariant (e.g., R346T), later confirmed by experimental studies.

Building on this knowledge graph, we developed the COVID-19 variant risk watcher (CVRW), an early-warning system that integrates graph-derived features with retrieval-augmented generation (RAG) to enhance GPT-4o in forecasting the WHO variant classification [variant under monitoring (VUM), variant of interest (VOI), or variant of concern (VOC)]. By retrieving mutation–function relationships and sentence-level evidence from CoVAR-KG, RAG injects domain knowledge beyond the model’s native context window, markedly improving accuracy compared to general-purpose language model. Furthermore, by linking mutation impact data to epitope mapping and antigenic drift models, our graph guides rational vaccine design and rapid update of immunogens against emerging strains. Finally, coupling the graph with retrieval-augmented generation enables rapid, in-depth functional analyses, thus empowering timely vaccine updates, therapeutic design, and informed clinical decision-making against evolving SARS-CoV-2 threats.

## 2 Materials and methods

### 2.1 Collection of COVID-19 literature

We constructed a comprehensive COVID-19 literature corpus by integrating PubMed and the Allen Institute’s COVID-19 repository. First, we retrieved 439,724 SARS-CoV-2–related PubMed identifier (PMIDs) (as of August 2024) *via* PubMed’s “Clinical Queries” COVID-19 filter, which encompasses nine thematic categories—general, mechanism, transmission, diagnosis, treatment, prevention, case reports, forecasting, and long COVID. This targeted approach ensures coverage of both foundational virology research and clinically oriented studies. Each PMID was annotated with metadata (title, abstract, MeSH terms, publication date) to support downstream classification and entity recognition.

Next, we matched these PMIDs to the COVID-19 full-text collection (June 2022 release), which aggregates coronavirus research dating back to early 2020. This step yielded 207,878 complete articles, providing access to detailed methods, results, and supplementary data. To maintain currency, we subsequently ingested newly published studies from PubMed and Europe PubMed Central (PMC), bringing our corpus to 340,904 full-text articles. All articles underwent standardized preprocessing—pdf-to-text conversion, section segmentation, and sentence tokenization—followed by automated entity extraction using our fine-tuned biomedical language model. This robust, up-to-date dataset underpins our large-scale knowledge extraction pipeline and the construction of a high-coverage SARS-CoV-2 knowledge graph.

### 2.2 Knowledge pattern induction

The COVID-19 literature forms a sprawling semantic network of molecular mechanisms and clinical observations, where entities—from spike-protein mutations to patient symptoms—interact through complex, condition-dependent relationships. To bridge basic virology and clinical practice, we adopted an inductive “Knowledge Pattern” approach: we systematically annotated a representative corpus (119 molecular studies, seven vaccine trials, and five clinical guidelines), extracting entities, relationships, and contextual conditions into subject–predicate–object triples. Recurring triples with similar syntactic structures and semantic roles were then clustered into distinct patterns, each capturing a coherent biological or clinical event.

This process yielded four molecular-level patterns—biochemical reactions, vaccine interactions, serum neutralization, and monoclonal-antibody binding—and five clinical-level patterns—symptomatology, diagnostics, treatments, indicators, and risk factors (Fig. S1). For example, the biochemical reaction pattern unites variant-specific kinetic parameters [e.g., angiotensin-converting enzyme 2 (ACE2) binding affinity, replication rate] under a unified schema for infectivity and pathogenicity. The vaccine interaction pattern formalizes statements such as “Variant X reduces neutralization by Vaccine Y,” while the serum and monoclonal antibody patterns capture analogous resistance or sensitivity assertions. On the clinical side, the diagnostic and treatment patterns encode method–disease and intervention–outcome relationships, respectively, and the indicator and risk factor patterns distill criteria for disease progression and patient vulnerability.

By encoding both molecular insights and patient-centered findings into standardized patterns, we create a shared semantic scaffold that directly links fundamental virology with clinical decision points. These patterns not only guide automated extraction of high-value knowledge from new publications but also enable the knowledge graph to support end-to-end queries—such as tracing how a spike-protein substitution alters viral kinetics, affects antibody neutralization, and ultimately informs treatment guidelines or vaccine updates. This integrative framework closes the gap between bench and bedside, empowering rapid translation of SARS-CoV-2 research into actionable clinical intelligence. The structured representation also supports integration of mutations from newly emerging variants and approximate reasoning about potential combinatorial effects via recurring knowledge motifs.

### 2.3 Construction of a curated, annotated corpus for model fine-tuning

We derived a high-quality annotation corpus from our SARS-CoV-2 literature repository (PubMed + COVID-19), isolating full texts and splitting them into individual sentences. A team of biomedical specialists was assembled and trained in our nine predefined knowledge patterns and triplet annotation protocol. Annotators—recruited for their expertise in virology, immunology, and clinical medicine—completed a 50-sentence qualification test (≥ 90 % accuracy) before proceeding to full annotation.

In the annotation phase, each sentence was labeled for the presence or absence of specific patterns, and positive examples were further decomposed into subject–predicate–object triples. Junior and senior annotators worked in a cross-review system, with any conflicts adjudicated by domain experts to maintain > 90 % overall accuracy. Throughout, we provided detailed glossaries, live Q & A support, and a comprehensive guidebook. The resulting dataset comprises 7,717 sentences annotated for pattern detection and 10,667 richly labeled triplets for entity and relation extraction. This rigorously curated corpus underpins our fine-tuning of large-scale language models for precise, domain-specific knowledge graph construction.

### 2.4 Knowledge pattern classification and knowledge extraction with LLMs

We adopted a full-parameter fine-tuning strategy enhanced by chain-of-thought (CoT) reasoning to tailor a language model for COVID-19 knowledge tasks. Beginning with a manually annotated corpus that captures nine distinct knowledge patterns and their associated triplets, we generated detailed reasoning chains—stepwise deductions that clarify the logic behind each annotation. These chains distilled complex scientific context into structured rationales, guiding the model’s comprehension of molecular and clinical relationships.

Using these CoT rationales, we fine-tuned a compact language model to inherit robust reasoning capabilities from a larger GPT-4o–class system. During training, the model learned to classify sentences into the predefined knowledge patterns and to extract subject–predicate–object triples with high precision. The structured prompts for rationale generation are illustrated in Fig. S2, while the prompts driving entity recognition and relationship extraction appear in Fig. S3.

We evaluated performance on held-out data, measuring both classification accuracy across nine COVID-19 patterns and precision/recall in triple extraction (entity recognition plus relation identification). The CoT-enhanced model achieved marked improvements over a baseline fine-tuned without rationales, demonstrating that integrating explicit reasoning chains streamlines complex knowledge extraction and elevates both efficiency and accuracy in domain-specific natural language processing (NLP) tasks.

### 2.5 Entity disambiguation

To resolve ambiguous entity names, we normalize all terms by lowercasing and stripping punctuation, then attempt exact matches against a curated dictionary of canonical labels. When no direct match exists, we compute pairwise similarity scores using Python’s Sequence Matcher, assigning each term to the dictionary entry with the highest score above a defined threshold. To capture deeper semantic relationships, we further leverage spaCy’s word embeddings: by measuring cosine similarity between entity vectors and dictionary labels, we refine assignments where string-based matching proves insufficient. This hybrid approach—combining deterministic lookup with both syntactic and semantic similarity—ensures accurate mapping of diverse nomenclature to standardized terminology.

### 2.6 Variant risk prediction with a knowledge graph–enhanced LLM using retrieval-augmented generation

We developed the CVRW to forecast the risk of emerging SARS-CoV-2 lineages by combining GPT-4o with retrieval augmented generation. The system links three steps: knowledge extraction, graph construction, and retrieval guided reasoning. A fine-tuned open-source language model first extracts mutation level triples from the scientific literature as described in Section 2.4. These triples are organized into a structured COVID-19 knowledge graph in Neo4j, which serves as the evidence base for retrieval. For a given variant, CVRW identifies salient features such as spike protein substitutions, immune evasion metrics, receptor binding affinities, and reported vaccine escape profiles. It then traverses the graph to collect the sentences that support these features, ranks the sentences by relevance and recency, and injects the selected evidence into structured prompt templates for GPT-4o. Only the original sentences that anchor the triples are provided, not full paragraphs or documents, which keeps the context compact, preserves traceability, and reduces unsupported outputs. Typically, 25 to 35 triples are retrieved per variant to balance coverage with inference speed. Prompts are organized for interpretability by presenting mutation level evidence first, followed by an integrative variant level summary, with each fact explicitly labeled by PubMed identifiers. This design concentrates the most informative facts in the model input, maintains token efficiency, strengthens factual grounding, limits hallucinations, and enables scalable and interpretable predictions of variant risk.

Each mutation node in the graph stores literature-derived evidence on phenotypic effects such as transmissibility, immune evasion, and therapeutic resistance. For newly emerging variants not yet represented in the graph, the system gathers the annotated effects of constituent mutations and composes a structured prompt. The language model then reasons in two steps: it first summarizes the known effects of individual substitutions and then integrates them to approximate variant behavior. Although the current graph does not explicitly encode epistasis with weighted edges, aggregation of mutation level evidence enables the model to capture some combinatorial patterns in a heuristic manner.

Temporal information is incorporated during retrieval so that recent studies receive higher weight when sources overlap or conflict. Publication dates are included to ensure that summaries reflect the current state of knowledge while retaining earlier reports for context. For example, on Pfizer–BioNTech vaccine effectiveness against B.1.617, early studies [27], [28] reported maintained protection, whereas later studies [29], [30] observed reduced neutralization. The retrieval stage prioritizes the latter, yielding summaries that align with the most recent evidence.

## 3 Results

### 3.1 Integrative knowledge patterns illuminate COVID-19 biology and clinical applications

**Fig. 1:**
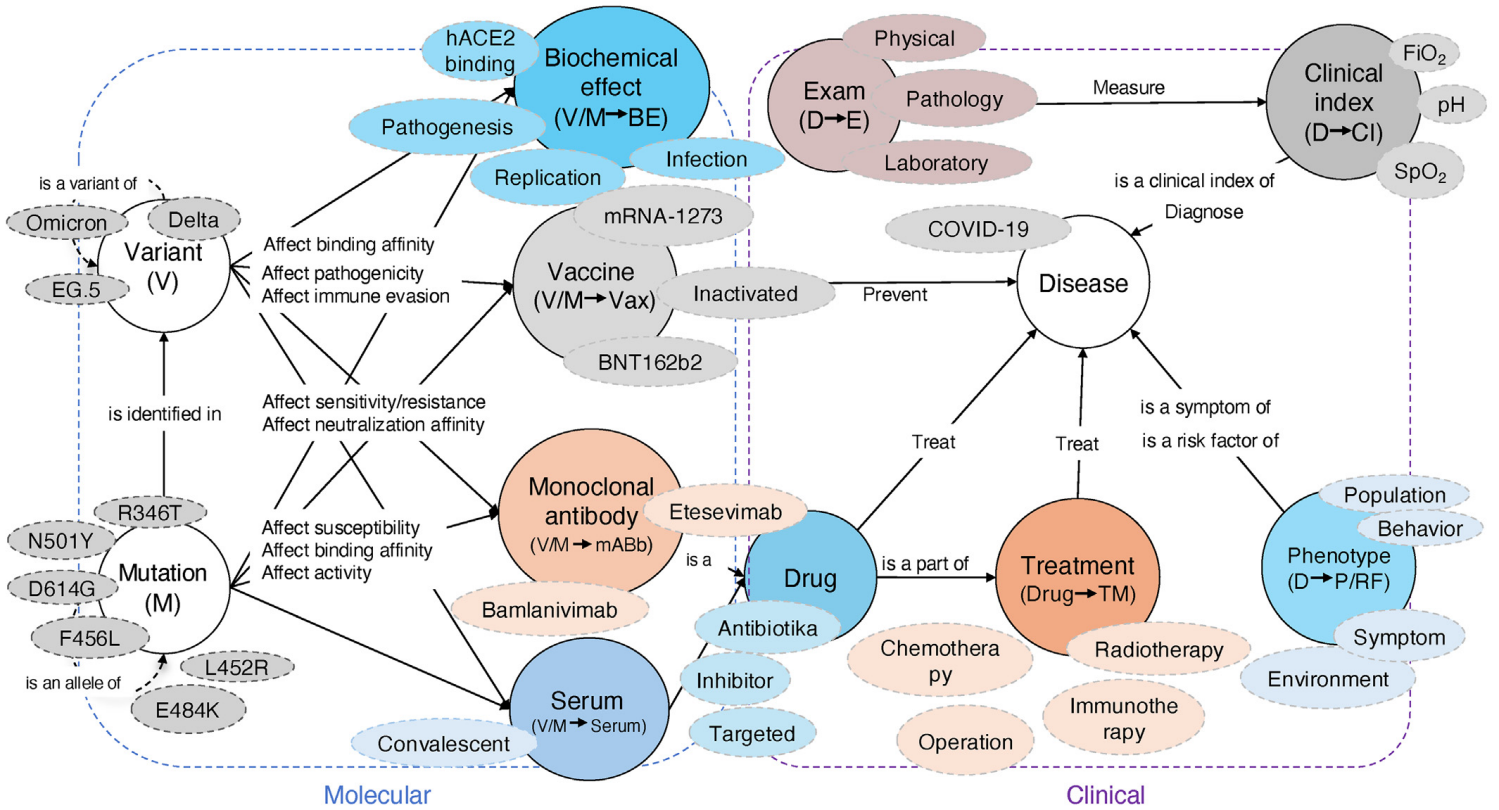
Schema of the COVID-19 molecular mechanisms and clinical outcomes knowledge graph. A unified schema organized into nine interconnected knowledge patterns that link viral genotype to patient phenotype. Variant (V): Major SARS-CoV-2 lineages (e.g., Delta, Omicron, EG.5) and defining spike mutations (R346T, N501Y, D614G). Mutation (M): Individual amino-acid substitutions driving viral entry, immune evasion, and altered function. Biochemical effect (V/M → BE): Molecular consequences (ACE2 affinity, replication kinetics, infectivity). Vaccine (V/M → Vax): Impact of genetic variation on vaccine efficacy (mRNA-1273, BNT162b2, inactivated). Monoclonal antibody (V/M → mAb): Neutralization profiles against therapeutic antibodies (Bamlanivimab, Etesevimab). Serum (V/M → Serum): Efficacy of convalescent plasma and polyclonal sera. Exam (D→E): Associations between disease phenotypes and laboratory diagnostics. Clinical index (D→CI): Patient parameters (SpO₂, FiO₂, pH) mapped to severity and trajectory. Therapeutics (Drug→TM): Pharmacological interventions linked to clinical outcomes. Phenotype (D→P/RF): Clinical phenotypes (Symptoms) and host or environmental risk factors (Population, Behavior) associated with disease occurrence, progression, or outcomes. Abbreviations: ACE2, angiotensin-converting enzyme 2; COVID-19, coronavirus disease 2019; SARS-CoV-2, severe acute respiratory syndrome coronavirus 2.

The COVID-19 Molecular Function and Clinical Diagnosis Knowledge Graph is built on nine distinct knowledge patterns rather than a collection of isolated triples. By grouping entities—ranging from viral variants, specific mutations and biochemical pathways to monoclonal antibodies, convalescent sera, vaccines, therapeutics, symptoms, risk factors and diagnostic markers—into these overarching patterns, the schema mirrors the multidimensional landscape of coronavirus research (Fig. 1). Each pattern represents a core axis of inquiry, seamlessly uniting molecular insights with clinical observations in a single, coherent framework.

This pattern-driven model forges direct links between foundational and applied studies. The “Variant–Mechanism” pattern, for instance, traces how spike-protein mutations reshape viral entry, while the “Therapeutic Response” pattern connects antibody binding affinities and drug efficacy metrics to patient outcomes. Rather than cataloguing isolated facts, the graph captures the dynamic interplay among mutation biology, diagnostic methods and treatment protocols.

By distilling the graph into reusable knowledge patterns, the schema empowers researchers to traverse from a mutation of interest through its biochemical reactions and mechanistic effects to clinical trial data and diagnostic marker studies. This unified architecture accelerates data integration, fuels hypothesis generation and lays a scalable foundation for the next wave of COVID-19 investigations.

This pattern-based framework enables seamless traversal from molecular alterations through functional consequences to clinical endpoints, supporting scalable integration and hypothesis generation across COVID-19 research domains.

### 3.2 Comprehensive annotation and large-scale extraction of COVID-19 knowledge patterns

To lay a rigorous foundation for our COVID-19 knowledge graph, we began by meticulously curating 2,562 peer-reviewed articles. Domain experts distilled 10,667 high-confidence triplets that capture both molecular biology—ranging from monoclonal antibodies and enzymatic reaction networks to serum biomarkers and vaccine constructs—and critical clinical insights, including risk factors, diagnostic features, symptom profiles and treatment modalities (Table 1). This manually vetted corpus spans 3,554 unique entities and 50 relationship types, ensuring that every node and edge reflects real-world expertise.

**Table 1:** Manual curation and LLM-driven extraction of COVID-19 knowledge.

Knowledge pattern	Manual annotation (n)	LLM inference (n)
Molecular–Antibody	1,508	21,515
Molecular–Reaction	417	21,433
Molecular–Serum	1,232	2,218
Molecular–Vaccine	1,663	7,947
Clinical–Risk factors	1,752	16,493
Clinical–Diagnosis	808	502
Clinical–Symptoms	1,181	34,013
Clinical–Indicators	643	35,529
Clinical–Treatment	1,463	156,236
Total	10,667	295,886

Abbreviations: LLM, large language model; angiotensin-converting enzyme 2; COVID-19, coronavirus disease 2019; SARS-CoV-2, severe acute respiratory syndrome coronavirus 2.

Building on this bedrock, we adopted a model-distillation strategy to rapidly expand our coverage. By fine-tuning a LLM on our curated dataset, the distilled network inferred over two million additional triplets in mere days. Following deduplication and consistency checks, we consolidated 295,886 novel, high-confidence relationships (Table 1). To enrich coverage further, we merged these machine-inferred triplets with 1,131,710 mutation–variant associations systematically drawn from curated biomedical repositories, yielding a hybrid dataset of more than 1.42 million structured relationships. These machine-extracted assertions embrace a wide spectrum of experimental protocols and population cohorts, and crucially, they encompass nearly all WHO-designated variants of concern and interest—achieving over 90 % representation of spike-protein mutations. This synergy of painstaking annotation and scalable inference preserves the accuracy of expert labels while dramatically accelerating knowledge discovery.

Entities in the LLM-inferred triplets are fully standardized. Relation text, however, remains heterogeneous and context dependent, often encoding mechanistic or functional nuance that resists complete normalization. To ensure interpretability and auditability, we expose the original source sentence for every inferred relation. This coupling of expert-curated training data with literature-scale inference maintains fidelity while enabling rapid knowledge acquisition.

Finally, we deployed an entity disambiguation pipeline to harmonize terminology and resolve semantic ambiguities across our enlarged graph. Variants, mutations, diseases and therapeutic agents are unified under consistent identifiers, safeguarding both recall and precision. By marrying expert-driven annotation with high-throughput model inference and robust normalization, our framework delivers a CoVAR-KG that is at once deeply reliable and exceptionally timely—poised to inform rapid insights into viral biology and clinical practice.

### 3.3 Model performance metrics

In the classification of knowledge patterns within COVID-19 literature, our fine-tuned Mistral-7B models—each trained on a bespoke, manually curated dataset—achieved state-of-the-art accuracy while preserving low-latency inference. The best performer, Mistral-7B-Instruct-v0.2, reached an accuracy of 86.9 %, surpassing Llama3-8B (85.8 %) and PMC-LLaMA-13B (84.9 %). By incorporating high-quality annotations into the fine-tuning loop, we were able to reduce model uncertainty on rare pattern classes and trim inference time by approximately 15 % compared with larger 13B-parameter baselines, demonstrating an optimal trade-off between performance and speed.

Scalability of our approach was validated by extracting 1,630,831 sentences harboring target patterns from a 40 million-sentence corpus, underscoring both the throughput of our pipeline and the robustness of the fine-tuned classifiers in handling real-world, large-scale text.

For knowledge graph construction, we next evaluated entity recognition and triple extraction. In entity recognition, Mistral-7B-Instruct-v0.1 led with an F1-score of 0.881 (precision 0.906, recall 0.857), highlighting its ability to pinpoint domain entities with high confidence. In triple extraction, the same model secured an F1-score of 0.714 (precision 0.758, recall 0.675). Although precision remained uniformly high across variants, recall dipped on low-frequency relations, suggesting that future work should integrate data-augmentation or pattern-generalization strategies to bolster coverage without sacrificing precision.

Table 2 summarizes these results, illustrating how fine-tuning on carefully annotated examples yields both high accuracy and efficient inference—key requirements for downstream tasks such as rapid knowledge graph population and real-time information retrieval.

**Table 2:** Model performance on coronavirus disease 2019 knowledge mining tasks.

Task	Model	Accuracy	Precision	Recall	F1-score
Knowledge pattern Classification	**Mistral-7B-Instruct-v0.2**	**0.869**	**0.829**	**0.776**	**0.801**
	Llama3-8B	0.858	0.803	0.772	0.787
	PMC-LLaMA-13B	0.849	0.811	0.726	0.776
	Meditron-7B	0.846	0.798	0.734	0.765
Entity recognition	Llama3-8B	−	0.876	0.828	0.852
	**Mistral-7B-Instruct-v0.1**	−	**0.906**	**0.857**	**0.881**
	Mistral-7B-v0.2	−	0.895	0.858	0.876
Triple extraction	Llama3-8B	−	0.742	0.651	0.694
	**Mistral-7B-Instruct-v0.1**	−	**0.758**	**0.675**	**0.714**
	Mistral-7B-v0.2	−	0.698	0.635	0.665

Note: “–” indicates that Accuracy is not applicable for span-based tasks; all scores were computed on held-out test sets; bold values indicate the best-performing model for each task.

### 3.4 Applications of the knowledge graph

#### 3.4.1 Linking mutational hotspots to functional outcomes

**Fig. 2:**
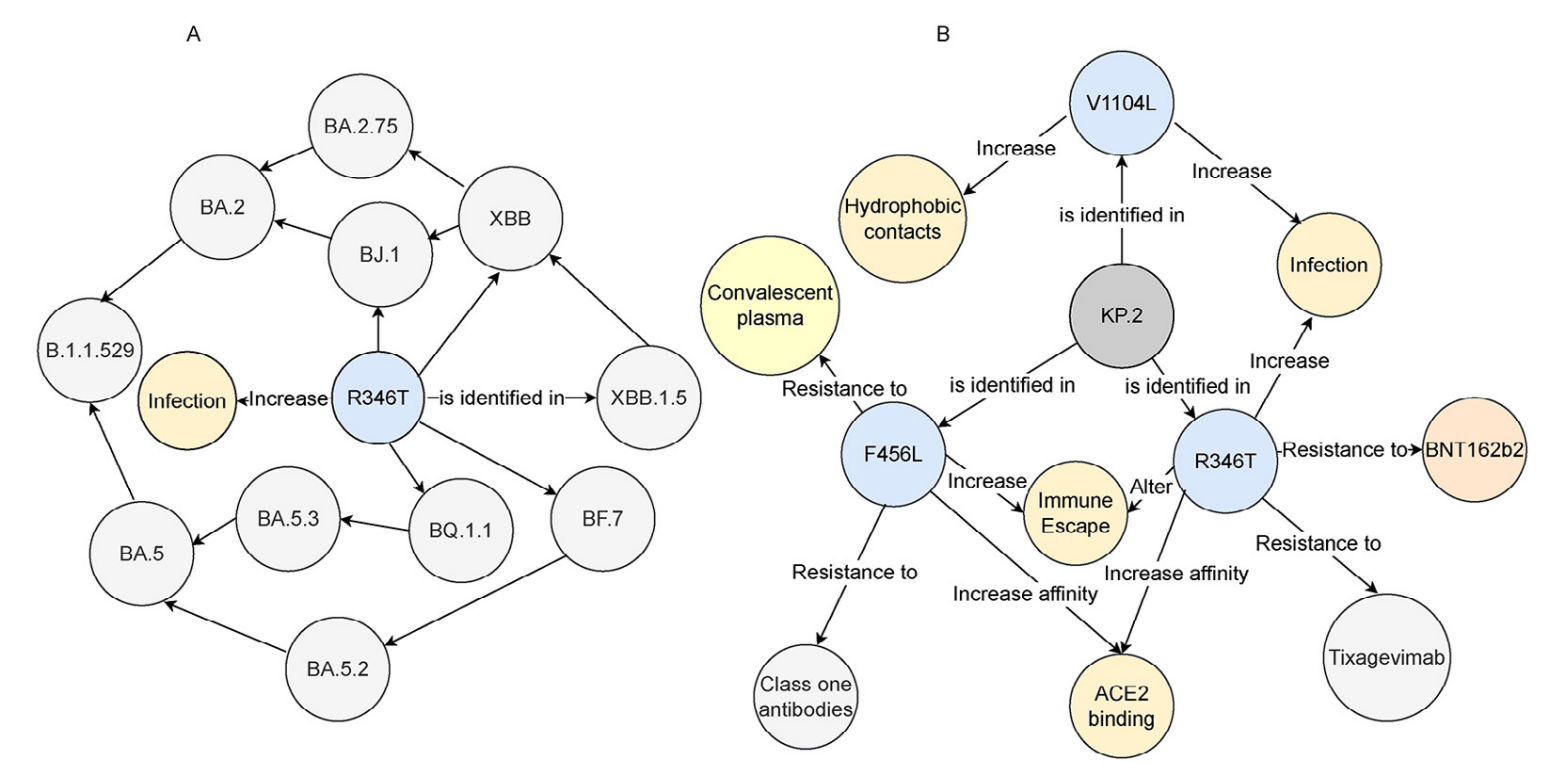
Forecasting high-risk mutation combinations and functional outcomes *via* knowledge graphs. A) Network of SARS-CoV-2 lineages linked by the R346T spike mutation. Lineages BJ.1, XBB, XBB.1.5, BF.7, and BQ.1.1 are shown. Red arrows trace inferred evolutionary paths; blue arrows mark independent emergence of R346T. B) Functional interaction map of key spike variants R346T, F456L, and V1104L. Functional interaction map connecting R346T, F456L, and V1104L to immune escape, ACE2 binding affinity, increased hydrophobic contacts within the spike stem, and resistance to neutralization. Edges represent literature-derived relationships structured by predefined knowledge patterns. Abbreviations: ACE2, angiotensin-converting enzyme 2; SARS-CoV-2, severe acute respiratory syndrome coronavirus 2.

Frequent spike-protein substitutions frequently forecast the phenotypic shifts of emerging SARS-CoV-2 lineages(Fig. 2B). For example, the R346T mutation—observed independently across multiple variants—enhances both host-cell entry and immune evasion [31]. Functional assays reveal that R346T strengthens ACE2 engagement while reducing neutralizing-antibody binding, highlighting its pivotal role in adaptive viral evolution.

The KP.2 sub-lineage of JN.1 exemplifies how synergistic mutation combinations drive rapid variant fitness gains. In addition to R346T [32], KP.2 carries F456L—linked to tighter ACE2 affinity and decreased antibody neutralization [33]—and V1104L, which strengthens hydrophobic contacts in the spike stem [34], stabilizes the prefusion trimer, and may enhance replicative capacity [35], [36]. Prior to February 2024, knowledge-graph-based analyses had already predicted that these three mutations would act together to boost transmissibility and immune escape. Subsequent experimental work confirmed significantly lower neutralization titers against KP.2 [37] and higher replication rates *in vitro*, validating the graph-based forecast (Fig. 2B).

By weaving structured virological data with literature-derived relationships, the knowledge graph supports retrieval-augmented LLM pipelines in rapidly uncovering critical mutation networks—such as the interplay among R346T, F456L, and V1104L—and distilling them into actionable insights. This approach empowers real-time evaluation of emerging variants’ transmissibility, pathogenicity, and antigenic drift, thereby accelerating the identification of high-risk lineages and guiding timely countermeasure development.

#### 3.4.2 Knowledge-graph–augmented early warning for SARS-CoV-2 variants

To test whether retrieval-augmented reasoning can reduce hallucinations and improve factual accuracy, we benchmarked CVRW against GPT-4o on an early warning task. Given the parental lineage designation and the set of novel spike-protein mutations of an emerging SARS-CoV-2 variant, the models were asked to predict whether the variant would later be classified as a VUM, VOI, or VOC.

Representative case studies illustrate CVRW’s interpretability. For the KP.2 lineage, CVRW correctly attributed elevated risk to S:R346T, S:F456L, and S:V1104L, mutations experimentally validated to enhance immune escape and spike stability [37], and classified KP.2 as a VUM. For KQ.1, which is not yet under official monitoring, CVRW highlighted S:T572I and S:R346T as potential drivers of increased transmissibility and immune evasion, warranting close follow-up. In contrast, GPT-4o either overlooked or misjudged the impact of these mutation combinations. These examples show how grounding predictions in curated mutation–function knowledge allows CVRW to deliver more reliable early warnings.

Table 3 summarizes results across 60 representative lineages. By integrating spike mutation effects, vaccine response signals, and transmission pathways, CVRW recalled 0.90 of VUM, VOI, and VOC cases with precision 0.70. GPT-4o often missed key mutations such as R346T and P681H or misjudged their combined effects, yielding recall 0.60 despite precision 0.85. For public health surveillance, broad coverage and early detection are essential, making CVRW’s operating point better suited for proactive monitoring. Its retrieval augmented reasoning over a curated mutation to function knowledge graph reduces unsupported outputs and improves predictive reliability. Supplementary Table S1 provides generation traces for representative variants that illustrate the system’s reasoning.

**Table 3:** Early warning performance of CVRW versus GPT-4o in SARS-CoV-2 variant risk prediction.

Model	Accuracy	Precision	Recall	F1-score
CVRW	0.783	0.718	0.933	0.812
GPT-4o	0.417	0.436	0.563	0.493

Abbreviation: CVRW, COVID-19 variant risk watcher; SARS-CoV-2, severe acute respiratory syndrome coronavirus 2.

All inputs to CVRW come exclusively from publicly available scientific literature and curated biomedical databases. The system processes no protected health information or personally identifiable information. Retrieved documents are cached only during processing and are never stored persistently, which aligns with established privacy standards. CVRW combines GPT-4o with RAG and uses a COVID-19 knowledge graph built by a fine-tuned open-source language model running on a standard graphics processing unit (GPU) cluster with four Nvidia A800 GPUs to extract mutation-level triples. Variant risk predictions are issued through GPT-4o API calls over the Neo4j knowledge graph, so local deployment of GPT-4o is not required. On standard cloud infrastructure, average end-to-end inference is 15–30 s per variant, with about 30 percent of time spent on knowledge graph queries and about 70 percent on GPT-4o processing. A typical query uses about 2,500 tokens across prompt and completion for five entities, which yields an estimated cost of 0.01–0.03 United States dollar (USD) per query at current GPT-4o pricing of 2.50 USD per million input tokens and 10 USD per million output tokens. These characteristics indicate that CVRW operates efficiently on common cloud resources while preserving the scalability and privacy safeguards needed for biosurveillance and public-sector deployment.

#### 3.4.3 Harnessing knowledge graphs for spike‐protein immunogenicity profiling

The functional annotation of SARS-CoV-2 spike variants can streamline the discovery of peptide antigens that elicit robust immune responses. By mapping variant sites onto structurally informed peptide segments, one can pinpoint residues that both drive immune escape and enhance receptor binding, guiding the selection of candidate antigenic peptides.

**Fig. 3:**
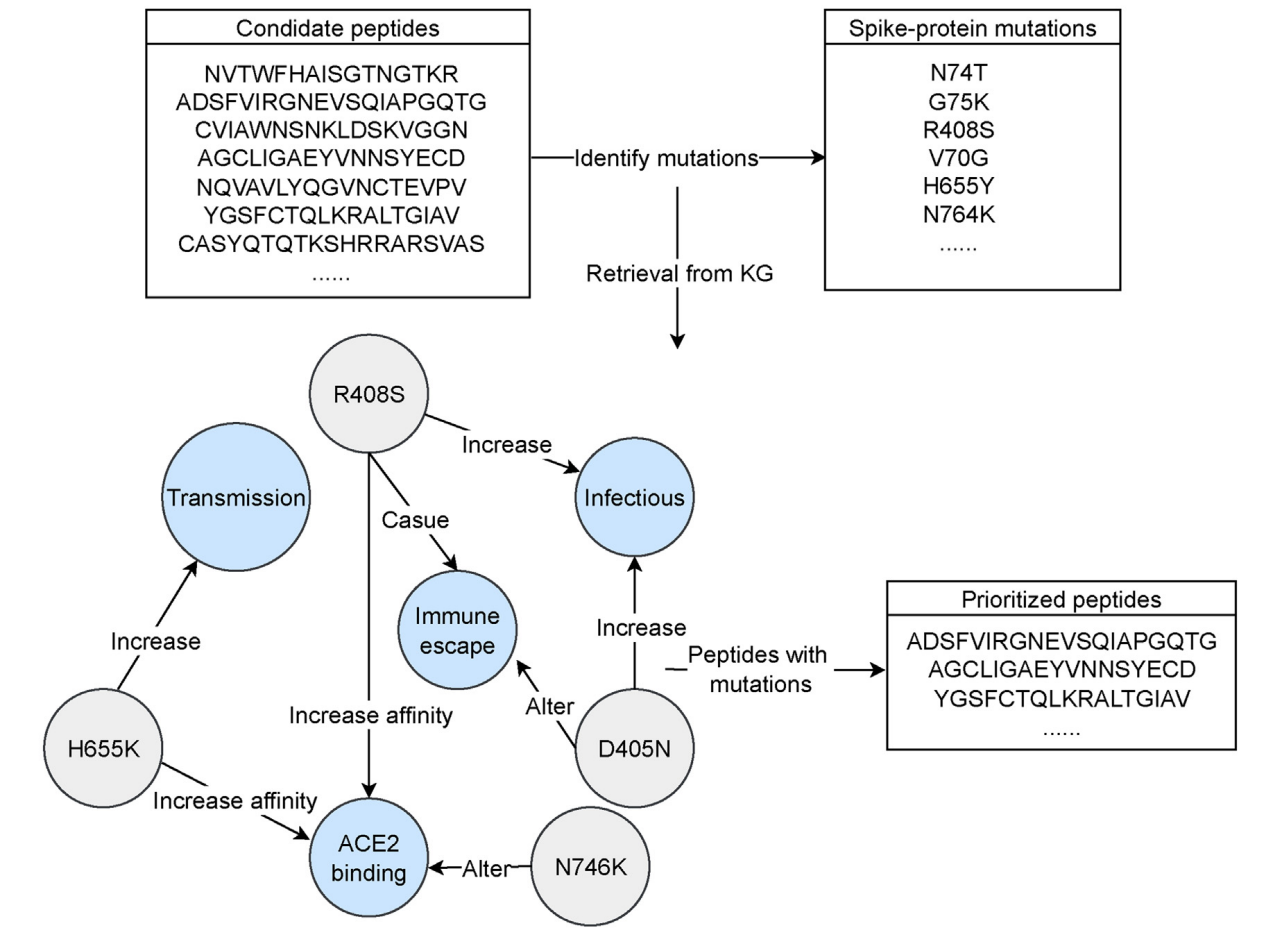
Knowledge graph–driven prioritization of spike-derived peptides *via* functional variant annotation. Functional mapping of 105 Omicron-subvariant spike mutations through our knowledge graph highlights residues that modulate ACE2 affinity and antibody escape. From these annotations, four candidate peptides (23b, P6, P16, P3) were selected for downstream immunogenicity testing. Abbreviations: ACE2, angiotensin-converting enzyme 2; KG, knowledge graph.

In one large‐scale effort, Wang and colleagues synthesized 97 overlapping peptides reflecting the BA.2 → BF.7 → XBB → JN.1 transmission cascade. Through knowledge‐graph–driven analysis, 105 variant positions were catalogued and functionally scored (Fig. 3). This revealed that G142D and P681H predominantly mediate antibody evasion, whereas N679K, G142D, P681H, K356T, and S50L contribute to heightened ACE2 affinity [38], [39], [40], [41], [42].

Leveraging these insights, four peptides—23b (CASYQTQTKSPHRARSVAS), P6 (QFCNDPFLDVYHKNNKSWME), P16 (ASVYAWNRTRISNCVAD), and P3 (VFRSSVLHLTQDLFLPF)—were prioritized for immunogenicity assays. Independent studies have since corroborated the antigenic potential of related motifs: QTQTNSPRRARSV exhibits exceptional surface exposure and may underlie both immune escape and ACE2 engagement, while NKSWME likewise shows strong antibody recognition, implicating it in viral evasion mechanisms [43]. These convergent findings validate the utility of knowledge‐graph frameworks in forecasting spike‐derived epitopes with high translational promise.

#### 3.4.4 Connecting molecular mutations with antiviral efficacy

To illustrate how our knowledge graph bridges detailed virological insights with real‐world treatment outcomes, we examined nirmatrelvir—the main protease (M^pro^) inhibitor in Paxlovid—against the Omicron BA.1 lineage. Clinical evidence confirms that nirmatrelvir maintains full antiviral potency against BA.1 infections, underscoring its continued therapeutic value [44].

**Fig. 4:**
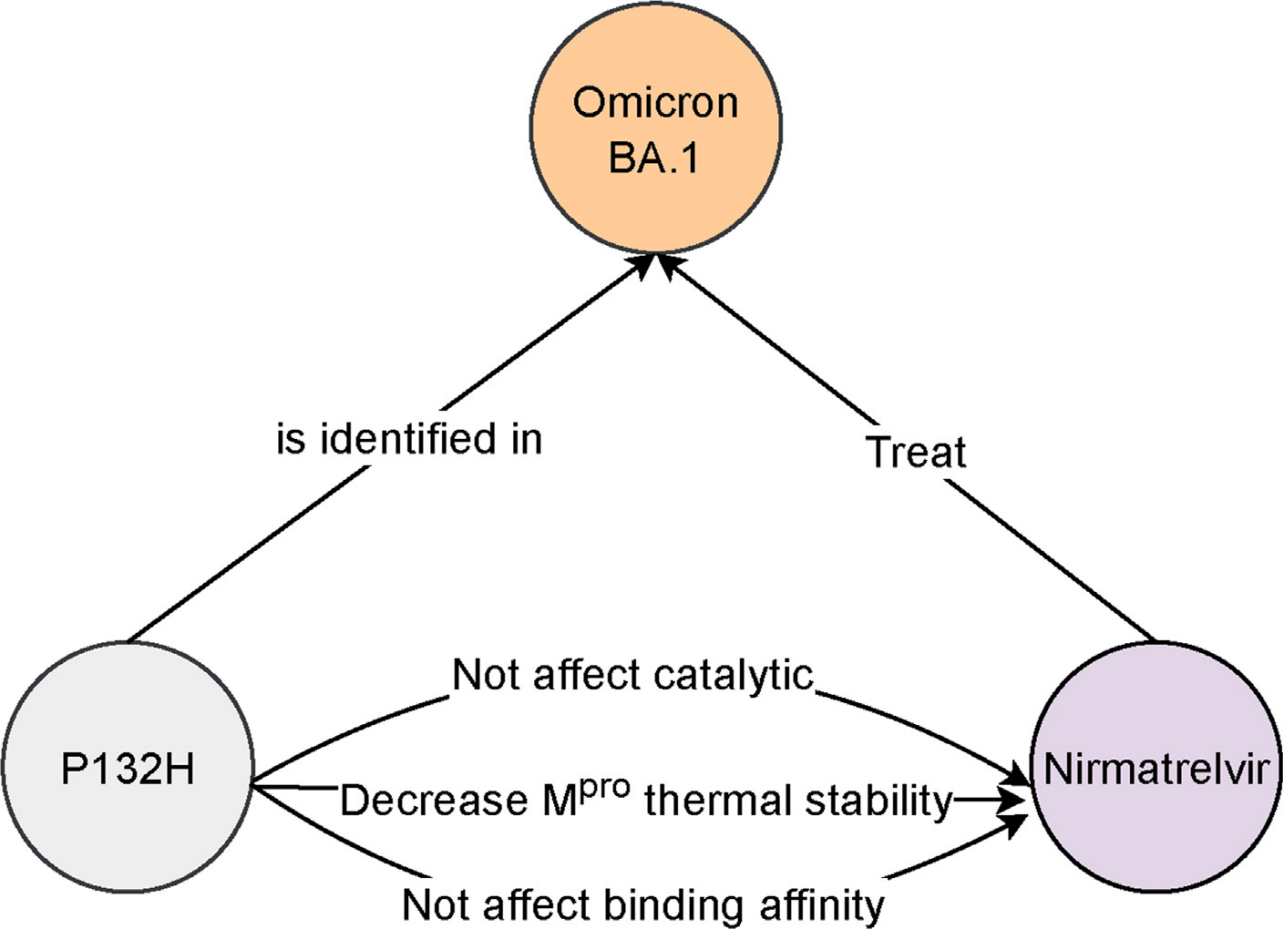
Mechanistic pathway from P132H mutation to nirmatrelvir efficacy in Omicron BA.1. A knowledge graph–integrated view of clinical, biochemical, and structural data reveals that the P132H substitution in the SARS-CoV-2 main protease (M^pro^) preserves enzymatic activity and inhibitor binding yet subtly alters protein stability—accounting for the sustained antiviral potency of nirmatrelvir. Abbreviation: SARS-CoV-2, severe acute respiratory syndrome coronavirus 2.

At the structural level, BA.1 carries a P132H substitution in nsp5, which modestly reduces the enzyme’s thermal stability without compromising its catalytic function or drug binding [45], [46], [47], [48]. Our graph encodes this chain of causality by linking the P132H mutation to both its biophysical effects and the unaltered efficacy of nirmatrelvir in clinical settings (Fig. 4).

By uniting mutation–function annotations with pharmacological outcomes, this case study highlights how knowledge‐graph frameworks can rapidly evaluate whether emerging variants threaten existing antivirals. Such mechanistic transparency accelerates decision‐making for public‐health authorities and informs the design of next‐generation therapies.

## 4 Discussion and conclusion

Taken together, this study presents three advances that deepen the understanding of SARS-CoV-2 biology and expand its biomedical applications. First, fine-tuning chain-of-thought reasoning on COVID-19–specific tasks substantially improved the model’s capacity to resolve complex virological and immunological questions. Second, we curated and annotated a comprehensive COVID-19 dataset that now serves as a benchmark resource for variant annotation and therapeutic assessment. Third, we extracted nine integrative knowledge patterns spanning molecular mechanisms and clinical outcomes, which streamline information extraction and promote standardized knowledge representation. Together, these advances enabled the construction of CoVAR-KG, a unified molecular-to-clinical knowledge graph that supports forecasting of emerging variants and refines antigenic-peptide screening pipelines for next-generation vaccine design.

Building upon CoVAR-KG, we developed CVRW, a knowledge-driven early-warning system that leverages RAG over graph-based evidence to forecast variant risks. Instead of reanalyzing raw literature, CVRW retrieves sentence-level evidence connected to mutation–function triples from the knowledge graph and injects it into GPT-based prompts for transparent, literature-grounded predictions.

Despite these advances, the pipeline exhibits lower accuracy in triplet extraction than in entity recognition, reflecting the intrinsic complexity of biological interactions. Temporal reasoning also remains limited. Although publication dates are used to weight recent evidence, the system does not yet perform longitudinal analysis across multimodal datasets. Achieving true temporal reasoning will require modeling dynamic trajectories and integrating time resolved data, which extend beyond a literature-centered framework. Future work will enrich the corpus with temporally diverse relationship examples, incorporate expert feedback loops, and develop advanced temporal graph models. These directions are expected to enhance relational reasoning, ensure temporal coherence, and ultimately strengthen knowledge-driven forecasting.
