# Supplementary material for: Leveraging knowledge graphs and large language models for integrating molecular variants and clinical insights in COVID-19 research
Source: Biosaf Health. 2025 Dec 20;8(1):71–9. doi: 10.1016/j.bsheal.2025.12.003 (PMC12931379; doi:10.1016/j.bsheal.2025.12.003)
Supplement: Supplementary Data 1 [file mmc1.docx]

# Supplementary Material


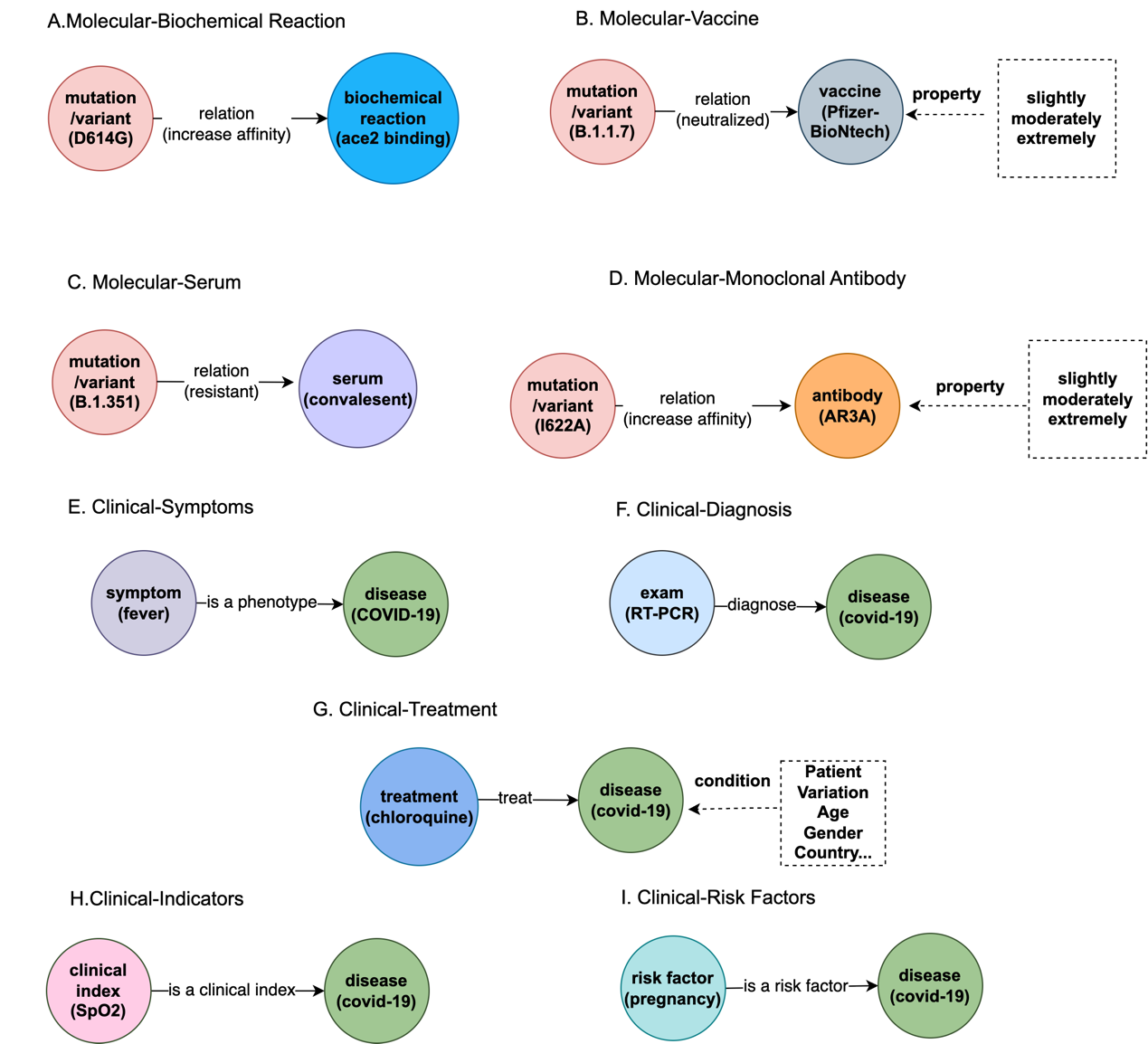


**Figure S1.** Knowledge Patterns Linking Molecular Mechanisms and Clinical Outcomes in COVID-19 Research. This figure illustrates nine distinct knowledge patterns derived from the COVID-19 literature, bridging molecular-level mechanisms with clinical practice:

(A) Molecular-Biochemical Reaction: Describes the impact of viral mutations/variants on biochemical reactions, such as increased ACE2 binding affinity by the D614G mutation.

(B) Molecular-Vaccine: Captures interactions between viral mutations/variants and vaccines, exemplified by the B.1.1.7 variant's neutralization resistance to Pfizer-BioNTech.

(C) Molecular-Serum: Represents mutation-induced resistance or sensitivity of convalescent serum, such as resistance by the B.1.351 variant.

(D) Molecular-Monoclonal Antibody: Details the binding affinity changes due to mutations, like increased affinity of the I622A variant to the AR3A antibody.

(E) Clinical-Symptoms: Encodes relationships between symptoms and the disease, illustrated by fever as a phenotype of COVID-19.

(F) Clinical-Diagnosis: Highlights diagnostic methods linked to the disease, such as RT-PCR for COVID-19 diagnosis.

(G) Clinical-Treatment: Describes treatment-disease relationships, with chloroquine as a treatment for COVID-19, modulated by patient conditions like age and gender.

(H) Clinical-Indicators: Presents clinical indices associated with the disease, such as SpO2 levels in COVID-19 patients.

(I) Clinical-Risk Factors: Identifies risk factors for disease progression, such as pregnancy increasing vulnerability to COVID-19.

These patterns create a unified semantic scaffold that integrates molecular insights with clinical decision-making, facilitating the translation of SARS-CoV-2 research into actionable clinical intelligence.


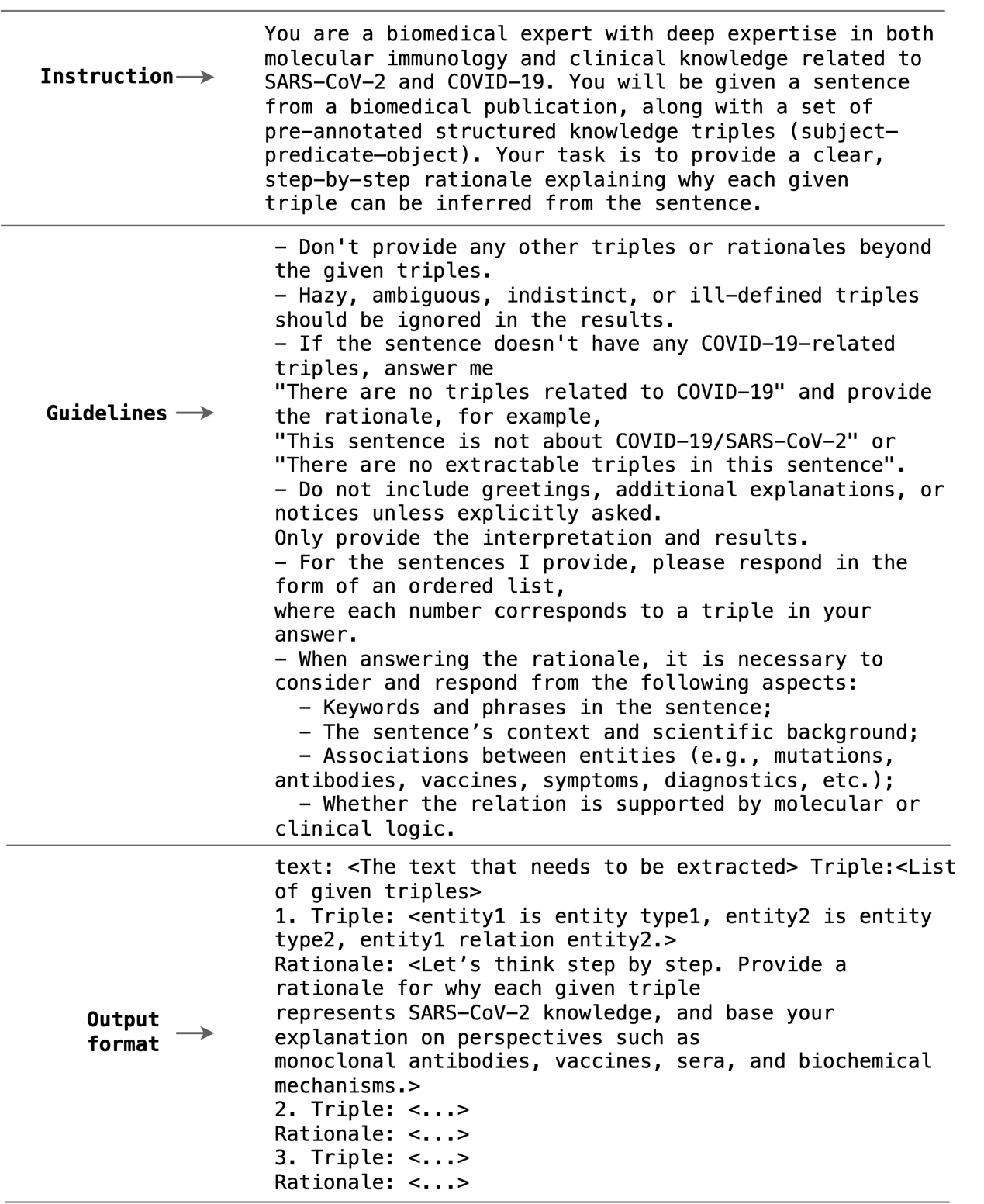


**Figure S2**. Prompt Template for Rationale Generation in SARS-CoV-2 Knowledge Extraction. This figure illustrates the structured prompt used to guide rationale generation in COVID-19 knowledge extraction. The prompt provides an input biomedical sentence alongside human-curated triples. The model is instructed to reason step-by-step and produce chain-of-thought explanations that justify the extracted triples.


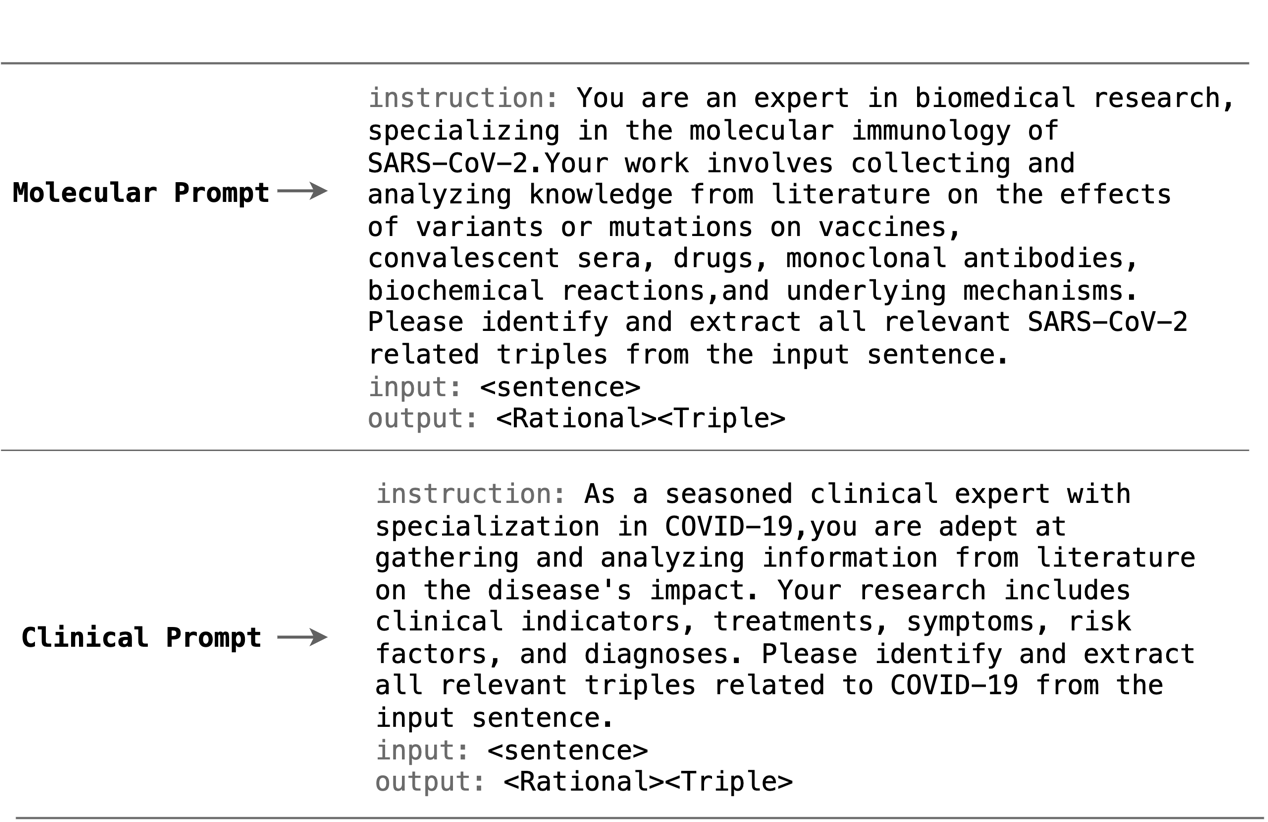


**Figure S3.** Prompt Template for Entity and Relation Extraction. This figure shows the streamlined prompt for extracting SARS-CoV-2–related triples from scientific text. It instructs the model to read each sentence, recognize biomedical entities, and identify their relationships to form structured knowledge triples. Two prompt variations are used: one for molecular-level extractions (e.g., mutation–vaccine interactions), and another for clinical-level extractions (e.g., symptom–diagnosis links).

**Table S1.** CVRW Predictions for Representative SARS-CoV-2 Variants.

| Variant | WHO Name | Label | CVRW Predict | GPT-4o Predict | CVRW Conclusion |
| --- | --- | --- | --- | --- | --- |
| B.1.617.2 | Delta | VOC/VOI/VUM | TRUE | TRUE | The B.1.617+F486P variant possesses genetic mutations that align with both VOI and VOC criteria. The original B.1.617 variant, known for its high transmissibility and potential immune escape, is further modified by the F486P mutation. This mutation could potentially enhance immune evasion and transmissibility by altering the receptor-binding domain's structure, affecting antibody binding and increasing ACE2 affinity. Given the existing knowledge about the B.1.617 variant's impact on public health, the additional mutation F486P suggests an increased risk of transmission and immune escape. Therefore, it is reasonable to classify B.1.617+F486P as a Variant of Concern (VOC) due to its potential to increase disease severity, transmissibility, and immune evasion, which could significantly impact public health measures, diagnostics, vaccines, and treatments. |
| B.1.351 | Beta | VOC/VOI/VUM | TRUE | TRUE | The presence of N501Y and K417N mutations suggests increased transmissibility and potential immune escape, which are critical factors in determining a variant's impact on public health. These mutations are known to enhance ACE2 receptor binding and reduce the efficacy of neutralizing antibodies, respectively. The epidemiological impact of these changes, such as causing significant community transmission or clusters, would further bolster its classification as a VOC. Additionally, if there is a demonstrated reduction in vaccine efficacy or treatment effectiveness, it would fulfill the criteria for VOC status. |
| JN.1 | Omicron | VOC/VOI/VUM | TRUE | FALSE | The variant's enhanced immune escape capability, due to the L455S mutation, and its altered interaction with the ACE2 receptor suggest that it could affect vaccine efficacy and transmissibility. Additionally, its resistance to monoclonal antibodies like Sotrovimab indicates a risk of therapeutic escape. These factors combined with the potential for increased community transmission and epidemiological impact suggest that BA.2.86.1+F19L+R3821K+L455S meets the criteria for a VOI, highlighting the need for continuous monitoring and evaluation to mitigate its impact on global public health. |
| LB.1 | Omicron | None of VOC/VOI/VUM | FALSE | FALSE | The R346T and F456L mutations are particularly associated with enhanced immune evasion and potential changes in ACE2 binding affinity, which could impact transmissibility and diagnostic or therapeutic escape. Although there are some indications of risk, such as increased immune escape and community transmission of JN.1, existing vaccines still provide protection to some extent. Given these factors, it is reasonable to classify this variant as a VUM, warranting continuous monitoring and assessment to gather more evidence on its epidemiological impact and to guide public health interventions. |
| BA.2.75 | Omicron | None of VOC/VOI/VUM | FALSE | FALSE | Based on this analysis, the COVID-19 variant <JN.1+T22N+F59S+F456L+Q493E+V1104L> can be classified as a Variant Under Monitoring (VUM). The mutations, particularly F456L and Q493E, suggest potential immune escape capabilities, which could pose a future risk. However, the full phenotypic and epidemiological impact is currently unclear, justifying enhanced monitoring. The variant aligns with the VUM criteria, as it shows genetic changes with suspected impacts on virus characteristics and immune escape, but lacks definitive evidence of significant epidemiological consequences. Enhanced surveillance and further research are warranted to assess its potential effects on public health. |
| XEC | Omicron | None of VOC/VOI/VUM | TRUE | FALSE | The mutations, particularly F456L and Q493E, suggest potential immune escape capabilities, which could pose a future risk. However, the full phenotypic and epidemiological impact is currently unclear, justifying enhanced monitoring. The variant aligns with the VUM criteria, as it shows genetic changes with suspected impacts on virus characteristics and immune escape, but lacks definitive evidence of significant epidemiological consequences. Enhanced surveillance and further research are warranted to assess its potential effects on public health. |
| BF.7 | Omicron | VOC/VOI/VUM | TRUE | FALSE | Although the specific mutations DEL30/32 and S33F require more data to understand their impact fully, the existing evidence suggests that this variant could pose a future risk. The current epidemiological data are not definitive enough to classify it as a VOI or VOC, but the potential for immune escape and resistance to treatment aligns with the criteria for a Variant Under Monitoring (VUM). Therefore, enhanced monitoring and further studies are warranted to determine its impact on public health. |
| KP.3 | Omicron | None of VOC/VOI/VUM | FALSE | FALSE | The F456L and Q493E mutations are particularly significant for their roles in immune evasion, impacting the effectiveness of neutralizing antibodies and vaccines. The variant also shows increased potential for immune escape, similar to other variants that have been monitored or classified as VOI or VOC. Given these characteristics, the variant aligns with the criteria for Variants Under Monitoring (VUM) due to its genetic changes and suspected impact on virus characteristics. Therefore, it should be subjected to enhanced monitoring to assess its epidemiological impact and potential risk to public health. |
| KP.2 | Omicron | None VOC/VOI/VUM | FALSE | FALSE | The R346T mutation contributes significantly to immune evasion and increased ACE2 affinity, while F456L further aids in immune escape, and V1104L may stabilize the spike protein, possibly affecting viral fitness. These mutations align with the VOI criteria, suggesting that this variant could impact global public health by increasing case numbers or reducing the effectiveness of current vaccines and treatments. Therefore, JN.1+S:R346T+S:F456L+S:V1104L has the potential to be classified as a VOI, warranting close monitoring and further investigation. |
| XBB.1.5 | Omicron | VOC/VOI/VUM | TRUE | TRUE | The XBB.1+F486P variant exhibits genetic characteristics that suggest a potential increase in transmissibility due to the F486P mutation, as it may enhance ACE2 binding. However, the extent of its effect on immune escape compared to XBB.1 is not significantly higher, indicating that its phenotypic impacts are not yet fully understood. This positions the variant as one that fits the definition of a Variant Under Monitoring (VUM). Enhanced monitoring is necessary to ascertain its impact on public health, particularly regarding transmissibility and the efficacy of current vaccines and treatments. Thus, given the evidence of its genetic changes and the potential to influence virus transmissibility and immune escape, XBB.1+F486P can be classified as a Variant Under Monitoring (VUM), necessitating ongoing surveillance to assess its impact on public health as more data becomes available. |
| B.1.429 | Epsilon | VOC/VOI/VUM | TRUE | TRUE | The mutations present in this variant are associated with increased transmissibility and possible immune escape, which are key criteria for VOIs. The presence of mutations in the spike protein, such as L452R, known for immune escape, and the combination of other mutations that could enhance viral replication and infectivity, suggest this variant could pose an emerging risk to global public health. However, further epidemiological data would be needed to confirm significant community transmission or clustering in multiple countries to fully establish its status as a VOI. |
| C.37 | Lambda | VOC/VOI/VUM | TRUE | FALSE | These features align with the VOI definition due to their potential impact on virus characteristics, including immune escape, and their ability to cause significant community transmission. The presence of mutations like L452Q and F490S, which are known for their roles in immune evasion and increased infectivity, further supports this classification. Therefore, based on the data from the literature and the genetic features of this variant, it is reasonable to predict that this specific COVID-19 variant could indeed be classified as a Variant of Interest (VOI). |
| B.1.621 | Mu | VOC/VOI/VUM | TRUE | FALSE | This is primarily due to the presence of key mutations such as N501Y and P681H, which are associated with increased transmissibility and immune escape. These mutations have been observed in other known variants of concern, highlighting their significance in the virus's ability to spread and evade immune responses. The literature supports the idea that these mutations enhance the virus's ability to bind to the ACE2 receptor and potentially evade neutralizing antibodies, which could lead to increased transmission rates and challenges in vaccine effectiveness. Given these characteristics, the variant could cause significant community transmission and impact public health measures, aligning with the criteria for a VOI. Further epidemiological data would be needed to confirm its spread and impact on global health, but the genetic profile suggests it warrants close monitoring. |
| B.1.617.1 | Kappa | VOC/VOI/VUM | TRUE | FALSE | The B.1.617 lineage, known for its significant mutations like L452R and E484Q, is associated with increased transmissibility and partial resistance to neutralizing antibodies. The additional mutations in this specific variant, such as Q1071H and I82S, might further influence its infectivity and immune escape abilities. Epidemiologically, the lineage has been linked to major outbreaks, notably in India, contributing to the surge in cases and posing a threat to public health. Although vaccines still offer protection against severe disease, the variant's ability to partially evade immune responses could lead to increased breakthrough infections. Thus, the B.1.617+I82S+S2312A+G1129C+Q1071H variant meets the criteria for a VOI, indicating it requires close monitoring and further investigation to understand its impact fully. |
| P.3 | Theta | VOC/VOI/VUM | TRUE | FALSE | The presence of mutations like E484K and P681H, which are known to affect transmissibility and immune escape, suggests that the variant could potentially increase the spread and severity of COVID-19. Additionally, the variant's potential impact on vaccine efficacy and diagnostic accuracy further supports its classification as a VOI. Given the evidence of its spread in multiple countries and the challenges it poses to current public health measures, it is reasonable to conclude that this variant meets the criteria to be considered a VOI. |
| BA.2.86 | Omicron | VOC/VOI/VUM | TRUE | FALSE | The combination of mutations, particularly R203K and G204R, along with others like P314L, contribute to increased transmissibility, immune escape, and potential changes in pathogenicity. The B.1.1.529 lineage is already recognized as the Omicron variant, which has had a significant epidemiological impact worldwide, demonstrating increased community transmission and immune escape. Therefore, this variant can be classified as a VOI due to its genetic changes affecting virus characteristics and its epidemiological impact, posing an emerging risk to global public health. |
| B.1.526 | Iota | VOC/VOI/VUM | TRUE | FALSE | The presence of mutations like D614G, T95I, and D253G, which are associated with increased transmissibility and immune escape, suggests this variant could impact virus spread and vaccine efficacy. The Q57H mutation further supports potential immune evasion capabilities. Consequently, this variant meets the criteria for a VOI due to its genetic changes that could affect virus characteristics and its potential epidemiological impact, warranting monitoring and further investigation. |
